# Expression of CD133, E-cadherin and WWOX in colorectal cancer and related analysis

**DOI:** 10.12669/pjms.332.11687

**Published:** 2017

**Authors:** Wenwen Sun, Jinxia Dou, Lin Zhang, Likui Qiao, Na Shen, Wenyuan Gao

**Affiliations:** 1 Wenwen Sun, Centre Lab, Tianjin Key Laboratory for Modern Drug Delivery & High-Efficiency, School of Pharmaceutical Science and Technology, Tianjin University, Tianjin 300072, China. Tianjin 4th Centre Hospital, Tianjin 300140, China; 2 Jinxia Dou, Centre Lab, Tianjin Key Laboratory for Modern Drug Delivery & High-Efficiency, School of Pharmaceutical Science and Technology, Tianjin University, Tianjin 300072, China. Tianjin 4th Centre Hospital, Tianjin 300140, China; 3 Lin Zhang, Centre Lab, Tianjin Key Laboratory for Modern Drug Delivery & High-Efficiency, School of Pharmaceutical Science and Technology, Tianjin University, Tianjin 300072, China. Tianjin 4th Centre Hospital, Tianjin 300140, China; 4 Likui Qiao, Department of Pathology, Tianjin Key Laboratory for Modern Drug Delivery & High-Efficiency, School of Pharmaceutical Science and Technology, Tianjin University, Tianjin 300072, China. Tianjin 4th Centre Hospital, Tianjin 300140, China; 5 Na Shen, Centre Lab, Tianjin Key Laboratory for Modern Drug Delivery & High-Efficiency, School of Pharmaceutical Science and Technology, Tianjin University, Tianjin 300072, China. Tianjin 4th Centre Hospital, Tianjin 300140, China; 6 Wenyuan Gao, Tianjin Key Laboratory for Modern Drug Delivery & High-Efficiency, School of Pharmaceutical Science and Technology, Tianjin University, Tianjin 300072, China

**Keywords:** Colorectal cancer, WWOX, CD133, E-cadherin

## Abstract

**Objective:**

To detect the expression of CD133, E-cadherin and WWOX in colorectal cancer, analyze the correlations and pathological significance of the biomarkers.

**Methods:**

Two hundred and ten patients with colorectal cancer treated surgically between January 2007 and December 2015 were analyzed retrospectively. All patients had pathologic specimens and integrated clinical data. Pathologic specimens were retrieved for immunohistochemical examination of the expressions of CD133, WWOX and E-cadherin. The clinical data of these patients including gender, age, tumor location, tumor size, tumor differentiation, invasion depth, hepatic metastases, lymphatic metastasis, UICC stage and recurrence of tumor were retrieved to investigate their demographics and clinical characteristics.

**Results:**

In 210 specimens of colorectal cancer, the positive expression rate of CD133, E-cadherin and WWOX was 61.9%, 40.5% and 41.9%, respectively. The expression of CD133, E-cadherin and WWOX was significantly correlated with lymphatic metastasis, hepatic metastases and UICC stage (p<0.05). The expression of CD133 was negatively correlated with WWOX and E-cadherin (p<0.05), and the expression of WWOX was positively correlated with E-cadherin in specimens (p<0.05).

**Conclusion:**

A detection of CD133, E-cadherin and WWOX can facilitate physicians in predicting the progression and prognosis of colorectal cancer.

## INTRODUCTION

Colorectal cancer, which accounts for 8% of all cancer deaths, is the third most common cancer in the world.^1^ Its treatment has gained importance in recent years. Currently, authors advocate that the recurrence and chemoresistance are the main problems for its treatment.^1^ Cancer stem cells (CSCs), also called tumor-initiating cells, have received high attention because they play a pivotal role in tumor invasion, metastasis and resistance to conventional therapy.^2^ Several molecular biomarkers have been used in clinical trials to demonstrate the existence of CSCs, among which CD133 is a valid marker for identifying cancer stem cells from fresh surgically resected colorectal cancer tissues to predict the early recurrence in colorectal cancer.^3^,^4^

In addition, the tumor suppressor gene WW domain-containing oxidoreductase (WWOX) is a 414-amino acid protein, its WW domain mediates protein-protein interactions and is essential for the signaling pathways used by tumor suppressors to inhibit tumor growth.^5^ Li suggests that WWOX may have potential clinical implications in bladder cancer therapy.^6^ In a study of 182 patients with leukemia of different types, Luo suggests WWOX has potential to be a good biomarker or predictor for leukemia therapy.^7^ WWOX can suppress stem cell properties in cancer stem cells, including self-renewal ability, differentiation potential, in vivo tumorigenic capability, high-level expression of stem cell genes and multidrug resistance.^5^

At the same time, the deregulation of the E-cadherin mediated cell adhesion system is correlated closely with cellular invasion, angiogenesis and metastatic progression in many cancers including colorectal cancer.^8^ The expression pattern of E-cadherin has been identified as an important prognostic factor for assessing tumor grade of colorectal cancer.^9^ Subsequently, we speculate that there may be close relationships between the expression of CD133, WWOX and E-cadherin in colorectal cancer tissues. However, studies focusing on the issues are not available in English literatures.

Therefore, the aim of the present study was to: 1) detect the expression of CD133, E-cadherin and WWOX in colorectal cancer; 2) evaluate the correlations between E-cadherin, CD133 and WWOX in colorectal cancer, facilitate physicians in predicting the progression and prognosis of the fatal disease.

## METHODS

Two hundred and ten patients with a biopsy-proven colorectal cancer treated surgically between January 2007 and December 2015 were analyzed retrospectively in the study. The inclusion criteria was: (1) the included patients underwent neither chemotherapy nor radiotherapy before surgery; (2) all the patients had pathologic specimens and integrated clinical data. Those patients with histories of other neoplasms were excluded. Pathologic specimens were retrieved for immunohistochemical examination of the expressions of CD133, WWOX and E-cadherin. The clinical data of these patients including gender, age, tumor location, tumor size, tumor differentiation, invasion depth, hepatic metastases, lymphatic metastasis, UICC stage and recurrence of tumor were retrieved to investigate their demographics and clinical characteristics. This study was approved by the Ethics Committee of our hospital.

In all cases immunohistochemical staining was performed on 5μm sections from paraffin-embedded fragments of the tumor specimen. Sections were deparaffinized using xylene, and rehydrated in ethanol baths using decreasing concentrations and finally in distilled water. Immunohistochemical technique and SP staining were performed. Positive and negative controls for each marker were used based on the manufacturer’s instructions.

The slides were examined and scored by two investigators independently. Immunohistochemical scoring was performed in a blind manner. In scoring expression of CD133, WWOX and E-cadherin, both the extent and intensity of immunopositivity were considered.^10^ The intensity was scored as follows: 0, negative; 1, weak; 2, moderate; 3, strong. The extent was scored based on the percentage of cells with positive staining: <10% is 1; 11%-50% is 2; 51%-75% is 3; >75% is 4. The final score was determined by multiplying the intensity and extent of positivity scores. The expression was considered positive when the scores were >1.^10^

The statistics was carried out using SPSS 21.0 (SPSS Inc., Chicago, IL, United States). The assessment of categorical variables was performed using Chi-squared test, and correlational analyses were conducted using Spearman rank correlation. A p value < 0.05 was considered as statistical significance.

## RESULTS

### The profile of the patients

The profile of the 210 patients is listed in Table-I. In 210 patients, 118 cases were male and 92 were female. Age ranged from 25 to 80 years old. Thirty six cases were right-sided colon cancer, 58 were left-sided colon cancer, 45 were sigmoid cancer, and 71 were rectal cancer. Sixty nine cases were high differentiated adenocarcinoma, 73 moderate differentiated adenocarcinoma and 68 low differentiated adenocarcinoma. In terms of invasion depth, 47, 53, 51 and 59 cases were T1, T2, T3 and T4, respectively. Lymphatic metastasis was detected in 112 cases, and no lymphatic metastasis in 98 cases. Hepatic metastasis was confirmed in 31 cases and no hepatic metastasis in 179 cases. Based on UICC stage, 42 cases were Stage I, 61 Stage II, 57 Stage III, and 50 Stage IV. The postoperative follow-up ranged from 8 to 60 months (Table-I).

**Table-I:** The expression of CD133, WWOX and E-cadherin and related pathological characteristics in 210 specimens of colorectal cancer.

*Variable*	*n*	*CD133*		*p*	*WWOX*		*p*	*E-cadherin*		*p*

		-	+		-	+		-	+
Gender				0.558			0.683			0.726
Male	118	47	71		70	48		69	49	
Female	92	33	59		52	40		56	36	
Age (year)				0.277			0.784			0.957
<60	86	29	57		49	37		51	35	
≥60	124	51	73		73	51		74	50	
Tumor location				0.129			0.337			0.938
Colon	139	58	81		84	55		83	56	
Rectum	71	22	49		38	33		42	29	
Tumor diameter				0.373			0.629			0.781
>5cm	121	43	78		72	49		73	48	
≤5cm	89	37	52		50	39		52	37	
Differentiation				0.170			0.005			0.076
High	69	32	37		30	39		38	31	
Moderate	73	27	46		44	29		39	34	
Low	68	21	47		48	20		48	20	
Invasion depth				0.712			0.020			0.561
T1	47	17	30		20	27		28	19	
T2	53	18	35		28	25		28	25	
T3	51	22	29		32	19		30	21	
T4	59	25	34		42	17		39	20	
Hepatic metastasis				0.005			0.049			0.028
Yes	31	5	26		23	8		24	7	
No	179	77	102		99	80		101	78	
Lymphatic metastasis				<0.001			<0.001			<0.001
Yes	112	19	93		83	29		80	32	
No	98	61	37		39	59		45	53	
UICC Stage				<0.001			0.003			<0.001
Stage I	42	30	12		18	24		6	34	
Stage II	61	18	43		29	32		31	27	
Stage III	57	18	39		38	19		42	15	
Stage IV	50	14	36		37	13		41	9	

### The relationship between the expression of CD133 and clinical pathology

In 210 cases, the positive expression of CD133 was found in 130 cases (61.9%), and negative expression in 80 cases (38.1%). There was no significant correlation between the expression of CD133 and age, gender, tumor size, tumor location, tumor differentiation and invasion depth (p>0.05), but there was significant correlation with lymphatic metastasis, hepatic metastasis and UICC stage (p<0.05).

### The relationship between the expression of E-cadherin and clinical pathology

In 210 cases, the positive expression of E-cadherin was found in 85 cases (40.5%), and negative expression in 125 cases (59.5%). There was no significant correlation between the expression of E-cadherin and age, gender, tumor size, tumor location, differentiation and invasion depth (p>0.05), but significant correlation with lymphatic metastasis, hepatic metastasis and UICC stage (p<0.05).

### The relationship between the expression of WWOX and clinical pathology

In 210 cases, the positive expression of E-cadherin was found in 88 cases (41.9%), and negative expression in 122 cases (58.1%). There was no significant correlation between the expression of WWOX and the age, gender, tumor size and tumor location (p>0.05), but significant correlation with lymphatic metastasis, hepatic metastasis, differentiation, invasion depth and UICC stage (p<0.05).

### The relationship between the expression of CD133, WWOX and E-cadherin and five-year survival rate

The five-year survival rate in CD133 positive expression group was 29.4%, and the negative group was 59.1%, there was significant difference between the two groups (p<0.05). However, in terms of E-cadherin, the five-year survival rate in positive expression group was 61.4% and in negative expression group was 26.3%(p<0.05). Similarly, the five-year survival rate in WWOX positive group was 55.8% and the negative group was 24.7% (p<0.05).

### The correlation between CD133, WWOX and E-cadherin

Spearman rank correlation analysis showed the expression of CD133 was negatively correlated with WWOX and E-cadherin in samples (p<0.05), with the expression of CD133 increased, the expression of WWOX and E-cadherin decreased. In addition, there was positive correlation between WWOX and E-cadherin in colorectal samples (p<0.05).

## DISCUSSION

In the current study, we found there was significant correlation between the expression of CD133 and lymphatic metastasis, hepatic metastasis and UICC stage, confirming the viewpoints of various studies. CD133, a pentaspan transmembrane protein, is regarded as a stem cell marker used to identify tumor initiating cells, especially in colorectal cancer.^4^ Kazama and colleagues analyzed CD133 expression in both primary tumors and lymph node metastases in stage III colorectal cancer by immunohistochemistry, found the patients with CD133-negative staining of either primary tumor or lymph node metastases had a higher overall survival rate than those with CD133-positive staining, indicating CD133-positive cancers may be more aggressive than CD133-negative ones during the process of colorectal cancer.^11^ In a study of forty patients with colorectal cancer, Kashihara also concluded that CD133 expression is correlated with poor prognosis in colorectal cancer patients.^12^

We also found there was significant correlation between the expression of E-cadherin and lymphatic metastasis, hepatic metastases and UICC stage, and the five-year survival rate in E-cadherin positive expression group was 61.4% and in negative expression group was 26.3%, this indicates that E-cadherin is an important marker of tumor aggressiveness. The viewpoint is consistent with Palaghia’s study.^8^ In addition, Palaghia found well and moderately differentiated tumors displayed an increased E-cadherin expression and poorly differentiated tumors a lack of E-cadherin expression.^8^ Ye also holds the similar viewpoint.^13^ In the present study, we found no significant correlation between the expression of E-cadherin and tumor differentiation. However, the p value was 0.076, indicating a similar trend with those previous studies. We think that a large sample study may be helpful in clarifying the issue.

Numerous studies demonstrate that WWOX gene is a tumor suppressor gene in various types of malignancies and either loss or reduction of the WWOX expression were found in variety of tumors.^14^ We found there was significant correlation between the expression of WWOX and lymphatic metastasis, hepatic metastases, differentiation, invasion depth and UICC stage, and the results are consistent with the previous studies,^14^,^15^ indicating WWOX is an important biomarker in predicting prognosis of colorectal cancer.

In addition, we found the five-year survival rate was significantly correlated with the expression of CD133, WWOX and E-cadherin, this demonstrates the close relationship between the three markers and the prognosis of colorectal cancer. At the same time, some authors have studied the correlations between these biomarkers. In a study of 110 colorectal cancer patients, Chen^16^ found a negative correlation between CD133 and E-cadherin, and a higher expression of CD133 is correlated with a lower expression of E-cadherin and worse prognosis in patients. In the current study, we found the expression of CD133 was negatively correlated with WWOX and E-cadherin, and a positive correlation between WWOX and E-cadherin in colorectal samples, this indicates a definite relationship between the three biomarkers in colorectal cancer, facilitating physicians to predict the progression and prognosis of colorectal cancer.

In short, we studied the expression of CD133, WWOX and E-cadherin in colorectal cancer and found the expression of CD133 was negatively correlated with WWOX and E-cadherin, and there was positive correlation between WWOX and E-cadherin. We believe this study may help physicians better predict the progression and prognosis of colorectal cancer.

### Limitations of the study

Although we found the close correlation between CD133, E-cadherin and WWOX in colorectal cancer, but the detailed molecular mechanism about the interactions between the three biomarkers is still unclear. Hence, more studies need to be performed in the future.
